# Universal screening of colorectal tumors for lynch syndrome: a survey of patient experiences and opinions

**DOI:** 10.1186/s13053-024-00290-8

**Published:** 2024-09-05

**Authors:** Alexander T. Petterson, Jennifer Garbarini, Maria J. Baker

**Affiliations:** 1 Genetic Counseling Program, Arcadia University, Glenside, PA USA; 2 CooperGenomics, Livingston, NJ 07039 USA; 3 Penn State Hershey Medical Center, Hershey, PA USA

**Keywords:** Lynch syndrome, Universal tumor screening, Informed consent, Service delivery models, Communication

## Abstract

**Background:**

Lynch syndrome represents the most common hereditary cause of both colorectal and endometrial cancer. It is caused by defects in mismatch repair genes, as well as *EPCAM*. Universal screening of colon tumors for Lynch syndrome via microsatellite instability (MSI) and/or immunohistochemistry (IHC) can identify patients and families at risk to develop further cancers and potentially impact surveillance and treatment options. The approach to implementation of universal screening, taking ethical considerations into account, is critical to its effectiveness, with patient perspectives providing valuable insight.

**Methods:**

Patients whose colon tumors underwent universal screening at Penn State Hershey Medical Center over a period of 2.5 years were mailed a survey on universal screening in 2017. Along with the survey, they received a recruitment letter and a summary explanation of research. The survey included both multiple choice and free-response questions that covered topics including respondent knowledge of Lynch syndrome, attitudes toward universal screening and experiences with the screening protocol as implemented.

**Results:**

Sixty-six of 297 possible patients (22.2%) responded to the survey, including 13 whose screening results raised concern for Lynch syndrome. 75.8% of respondents supported universal tumor screening without informed consent. 92.4% preferred receiving screening results regardless of outcome. Respondents described benefits to screening for themselves and their families.

**Conclusions:**

While broadly supporting universal tumor screening without informed consent, respondents also wanted more information shared about the screening policy, as well as their results. These patient preferences should be one of many factors considered when implementing universal screening and can also inform practices regarding both tumor profiling and universal genetic testing, which is becoming more prevalent.

## Background

Lynch syndrome increases the risk to develop both colorectal and endometrial cancer, as well as cancer of the stomach, ovary, hepatobiliary tract, urinary tract, small bowel, brain, sebaceous glands, and pancreas [1]. Lynch syndrome follows an autosomal dominant mode of inheritance and is due to pathogenic variants in the DNA mismatch repair (MMR) genes *MLH1*, *MSH2*, *MSH6*, and *PMS2*. Terminal deletions in the *EPCAM* gene, which is upstream of *MSH2* also result in Lynch syndrome [2].

The diagnosis of Lynch syndrome can alter medical management, decrease the incidence of cancer, and save lives. If an individual with metastatic cancer is identified to have Lynch syndrome and their tumor demonstrates defective MMR protein expression, they may be a candidate for immunotherapy with the potential for improved progression-free survival. Individuals with Lynch syndrome who do not have cancer can clarify their cancer risks and take appropriate steps to reduce those risks. Increased screening for colorectal cancer through earlier and more frequent colonoscopies can significantly increase survival rates and decrease both the incidence and mortality of colon cancer in individuals with Lynch syndrome [3, 4]. Endometrial biopsy, as well as the option of hysterectomy and bilateral salpingo-oophorectomy are options for some females with Lynch syndrome, depending on the gene involved, to address their risk for endometrial and ovarian cancer [5]. While various criteria based on personal and family cancer history have been used to identify patients with Lynch syndrome, numerous barriers to this approach have been identified [6–8]. As a result, it has been recommended that all patients diagnosed with colon cancer be screened and, if positive, be offered genetic counseling and/or genetic testing [2, 5, 9, 10].

The effectiveness of this universal Lynch screening depends on patient follow-up after the initial positive screen and whether those patients with a confirmed Lynch-associated variant share information with at-risk relatives to promote cascade testing. Prospective studies suggest that most patients feel equipped to cope with tumor testing results and that a majority of patients anticipate sharing results with family members prior to receiving their results [11, 12]. However, some patients have declined germline testing, leading to reduced effectiveness of universal tumor screening [13]. Having a dedicated genetic counselor follow up with patients whose screening results raise concern for Lynch syndrome has resulted in better uptake of testing and a higher overall detection rate [14, 15].

At the Penn State Hershey Medical Center, universal screening of all in-house, invasive colorectal tumor specimens for Lynch syndrome was implemented on May 1, 2014. Tumors are subjected to immunohistochemistry (IHC) testing for *MLH1*,* MSH2*,* MSH6*, and *PMS2*. If *MLH1* expression is absent, the tumor is then sequenced for presence of the *BRAF* V600E pathogenic variant, a common sporadic cause of colorectal cancer [16]. Patients whose tumors demonstrate absent expression for any of the four mismatch repair proteins and no *BRAF* V600E variant are contacted by a genetic counselor to discuss the potential concern for Lynch syndrome and offered an appointment for consideration of genetic testing. Any patient who cannot be reached by telephone is mailed a letter regarding their positive screening results and the potential implications for Lynch syndrome, with contact information for the Cancer Genetics Program included. Individuals who schedule a genetic counseling appointment have the opportunity to discuss and pursue germline testing of the appropriate gene (s) and/or a larger panel of genes to determine whether they have Lynch syndrome or possibly some other hereditary predisposition to cancer. Those identified to have a pathogenic variant are then managed per the National Comprehensive Cancer Network (NCCN) Guidelines^®^ and encouraged to discuss their results with at-risk family members.

While universal tumor screening is broadly supported within the medical community, there has been debate regarding whether informed consent is necessary for screening. Some argued that IHC screening may reveal information about a patient’s germline DNA and thus should require consent [17]. Others suggested that IHC screening is similar to other pathology testing such as hormone receptor testing in patients with breast cancer and thus does not require consent [18]. Clinicians have also had concerns that a consent process would create a barrier to screening and detection [19]. Some have suggested that an opt-out approach similar to that used in newborn screening may be a viable compromise to this ethical debate [20]. The preferences of oncology patients with regard to consent are less well documented in the literature, though in a recent survey of patients with colorectal cancer whose tumors had not undergone universal screening, a majority believed consent should be obtained [21].

This study aims to take a retrospective approach to evaluate patient opinions and experiences regarding the universal colorectal tumor screening policy at an academic medical center. Important topics include satisfaction with an implemented universal screening policy without informed consent, the reasons patients pursue or decline genetic counseling and genetic testing, sharing of test results with family members, and impact of screening and test results on patients and their families.

## Methods

### Participants

Potential study participants included patients whose colorectal tumors were screened for Lynch syndrome between May 1, 2014 and November 1, 2016 (inclusive) at the Penn State Hershey Medical Center. Recruitment was conducted by mailing a packet including a recruitment letter, a summary explanation of research, a paper copy of the survey, and instructions to fill out either the enclosed paper survey or the online version on the Penn State University Research Electronic Data Capture (REDCap) website. Study participants consisted of those individuals who completed the online survey or who completed and mailed the paper survey within 12 weeks of the postmark on the envelope containing the recruitment packet. As explained in the summary explanation of research, consent to participate was implied by return of a completed survey. Participants received a $10 gift card for Amazon.com as compensation for their time taken to complete the survey. Gift cards were mailed by an honest broker following receipt of the paper or online survey.

### Instrumentation

The survey consisted of both multiple-choice and free-response questions. There were 48 questions, though some questions only applied to a subset of participants. Some changes were made to the wording of questions in the online questionnaire due to the implementation of branching logic. For example, instructions from the paper copy of the survey such as, “Skip to question…” were removed since the branching logic removed any irrelevant questions.

### Data analysis

Descriptive statistics were used to describe the answers to multiple-choice questions. Free response questions were analyzed for themes and then classified according to those themes by the author and an advisor. Disagreements on theme classification were resolved via conversation, with the final decision made by the first author. Fisher’s Exact tests were used to determine whether respondents differed significantly from non-respondents. Fisher’s Exact test was also used to compare the opinions of different groups regarding universal screening for Lynch syndrome without informed consent.

## Results

### Participation

Sixty-six of 297 (22.2%) patients who were screened for Lynch syndrome responded to the survey. This included 10 of 28 overall (35.7%) who had genetic counseling and one who had not yet had a scheduled appointment, 9 of 26 (34.6%) who had germline genetic testing, and 3 of 7 (42.9%) who tested positive for Lynch syndrome. Four respondents who underwent germline testing reported having a variant of uncertain significance (VUS) and 2 reported normal results. Two participants responded online using REDCap while 64 returned the paper survey by mail. Paper surveys were copied into the REDCap system by an assistant and checked by at least one of the primary researchers.

### Demographics

The survey was sent to 156 males and 141 females. Of these, 42 (64.6%) respondents were female while 23 (35.4%) were male. Respondents reported a variety of educational backgrounds, with 27 of 65 (41.5%) reporting a college education or more and 18 of 65 (27.7%) having a high school diploma or less. Ages of respondents varied, with approximately one third (*n* = 22/64) being age 59 or younger and two thirds (*n* = 42/64) being age 60 or older. Table 1 provides further breakdown of respondent demographics.

**Table 1:** **Demographics**

Sex	*N*	Percentage
Male	23	35.4%
Female	42	64.6%
**Age**		
30–39	3	4.7%
40–49	4	6.3%
50–59	15	23.4%
60–69	22	34.4%
70–79	12	18.8%
80+	8	12.5%
**Marital Status**		
Married	44	67.7%
Single with partner	4	6.2%
No partner	2	3.1%
Widowed	10	15.4%
Divorced	5	7.7%
**Level of Education**		
Less than High School	1	1.5%
High School	17	26.2%
Some College or Technical School	20	30.8%
College	17	26.2%
Some Graduate or Professional School	5	7.7%
Graduate or Professional School	5	7.7%
**Racial Background** ^**a**^		
American Indian or Alaska Native	1	1.5%
Black or African American	2	3.1%
White or Caucasian	64	98.5%
**Estimated Household Income**		
<$25,000	6	10.7%
$25,000-$49,999	20	35.7%
$50,000–74,999	13	23.2%
$75,000-$99,999	5	8.9%
>$100,000	12	21.4%

^a^Participants could select multiple racial backgrounds

### Knowledge of Lynch syndrome and universal screening

**Fig. 1:**
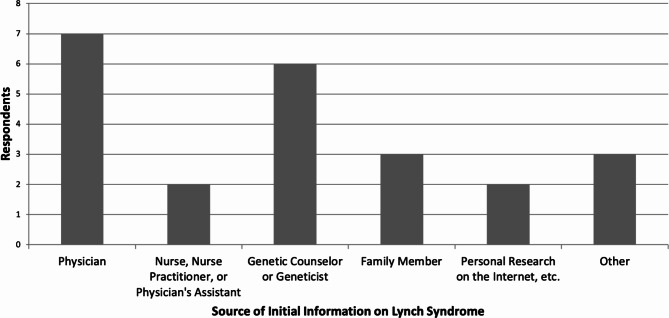
**Ways in which respondents first learned of Lynch syndrome.** Respondents who first learned of Lynch syndrome from a physician specified the physician was a colorectal surgeon, a gastroenterologist, or an oncologist. The three responses listed as “Other” were respondents who checked multiple responses on the paper copy of the survey. Three checked physician, two checked genetic counselor or geneticist, and one each checked personal research on the Internet, etc. and nurse, nurse practitioner, or physician’s assistant

Twenty-three of 66 (34.8%) respondents had previously heard of Lynch syndrome (Table 2). They learned from a variety of sources including medical professionals, family members, and the Internet (Fig. 1). Fourteen of 65 (21.5%) respondents had heard of the hospital’s universal screening program for Lynch syndrome. Twenty-two of 66 (33.3%) reported knowing their tumor had been screened for Lynch syndrome, and twelve of these (54.5%) reported being informed prior to screening.

**Table 2:** **Prior knowledge of Lynch syndrome and screening**

	Yes	No	Uncertain
Before receiving this survey, had you heard of Lynch syndrome, also known as Hereditary Non-Polyposis Colorectal Cancer Syndrome (HNPCC)?	23 (34.8%)	42 (63.6%)	1 (1.5%)
Before receiving this survey, did you know that your hospital had a universal screening policy for Lynch syndrome?	14 (21.5%)	49 (75.4%)	2 (3.1%)
Before receiving this survey, did you know that your tumor specimen was screened for Lynch syndrome?	22 (33.3%)	37 (56.1%)	7 (10.6%)

### Opinions on universal screening

Fifty respondents (75.8%) supported universal screening for Lynch syndrome without patient consent, eleven (16.7%) did not support it, and five (7.6%) were uncertain. The supporters included all respondents who had a pathogenic mutation associated with Lynch syndrome identified through universal screening. More than 20 respondents who supported screening endorsed each of several reasons suggested in the survey. The most endorsed response noted that consent should not be required because screening does not genetically diagnose Lynch syndrome. Those who did not support screening without consent were most concerned by lack of formal consent, though some were also concerned about out-of-pocket cost. They favored a variety of methods, both verbal and written, for consenting.

### Being informed of results

92.4% (*n* = 61/66) of participants believed that all patients whose tumors were screened should be informed of their screening results. A few (*n* = 4/66) believed that the current method under which negative results are not returned was acceptable, and one believed patients should decide when to receive results when they receive information prior to screening. 55.4% (*n* = 36/65) preferred to be informed by the physician who collected the sample. A minority (*n* = 17/65) preferred that a genetics professional inform them. The remainder preferred to be contacted by mail (*n* = 4/65), believed that multiple methods of contact would be acceptable (*n* = 6/65), or thought that the method of contact should depend on the screening result (*n* = 2/65).

### Pursuing genetic counseling

Thirteen of 62 respondents (21.0%) reported having universal screening results that showed a potential concern for Lynch syndrome, of whom 11 sought genetic counseling. Those who sought genetic counseling endorsed multiple reasons for having done so, with a majority endorsing the following: (1) understand why they developed colorectal cancer, (2) inform medical decisions, and (3) understand risks for children and other relatives. Respondents who did not seek genetic counseling felt they had too much going on at the time they were notified and were concerned about potential out-of-pocket costs of the appointment.

### Discussing genetic counseling and test results with family members

All 11 respondents who sought genetic counseling reported sharing their decisions with their families, and all had at least some supportive relatives with the majority (*n* = 9/11) reporting that all relatives were supportive. The three respondents who received positive genetic test results reported sharing those results with spouses and biological relatives.

### Decisions based on a positive diagnosis

The three participants confirmed to have Lynch syndrome reported using their test results to make a variety of life decisions. Two individuals opted for a more extensive risk-reducing colon resection and the third also modified their surgery plans. All three are getting more frequent colonoscopies and two report adding upper endoscopies to their screening regimens. One also began having annual urinalysis and physical and skin exams while another reported getting CEA blood tests and PET and CT scans. Two participants reported changing their exercise regimens and diets based on their Lynch syndrome diagnoses. One of these also reported starting a weight loss program, quitting tobacco, decreasing alcohol consumption, and decreasing sun exposure. This person reported feeling healthier and also had a polyp removed before it developed into a new cancer.

### Opinions on an information sheet sharing the universal screening policy

When asked, 82.3% (*n* = 51/62) of respondents believed an information sheet regarding the hospital’s universal screening program would be helpful. 52% (*n* = 26/50) believed that sheet should be provided after a confirmed diagnosis of colorectal cancer, but others endorsed receiving the information (*n* = 8/50) before a colonoscopy or after screening raised concern for Lynch syndrome (*n* = 12/50).

### Impressions of hospitals with universal screening policies

A majority (*n* = 55/63) of respondents would recommend treatment at a hospital with a universal screening policy, compared with one respondent who would not. For 90.6% (*n* = 58/64) of respondents, a universal screening policy reinforces confidence in the hospital’s ability to provide state-of-the-art care. 84.4% (*n* = 54/64) of respondents said there should also be a universal screening policy as part of endometrial cancer treatment. There were few negative responses to these three ideas (Table 3).

**Table 3:** **Impressions of hospitals with universal screening**

	Yes	No	Uncertain
Would you encourage friends or relatives to pursue their screening colonoscopies or colorectal surgery, if needed, at a hospital that has implemented a universal screening policy?	55 (87.3%)	1 (1.6%)	7 (11.1%)
Does the fact that your hospital has implemented a universal screening policy for Lynch syndrome reinforce your confidence in their ability to provide state-of-the-art medical care?	58 (90.6%)	3 (4.7%)	3 (4.7%)
Do you think hospitals should also screen all women diagnosed with uterine cancer for Lynch syndrome?	54 (84.4%)	0 (0%)	10 (15.6%)

### Impact of screening

**Fig. 2:**
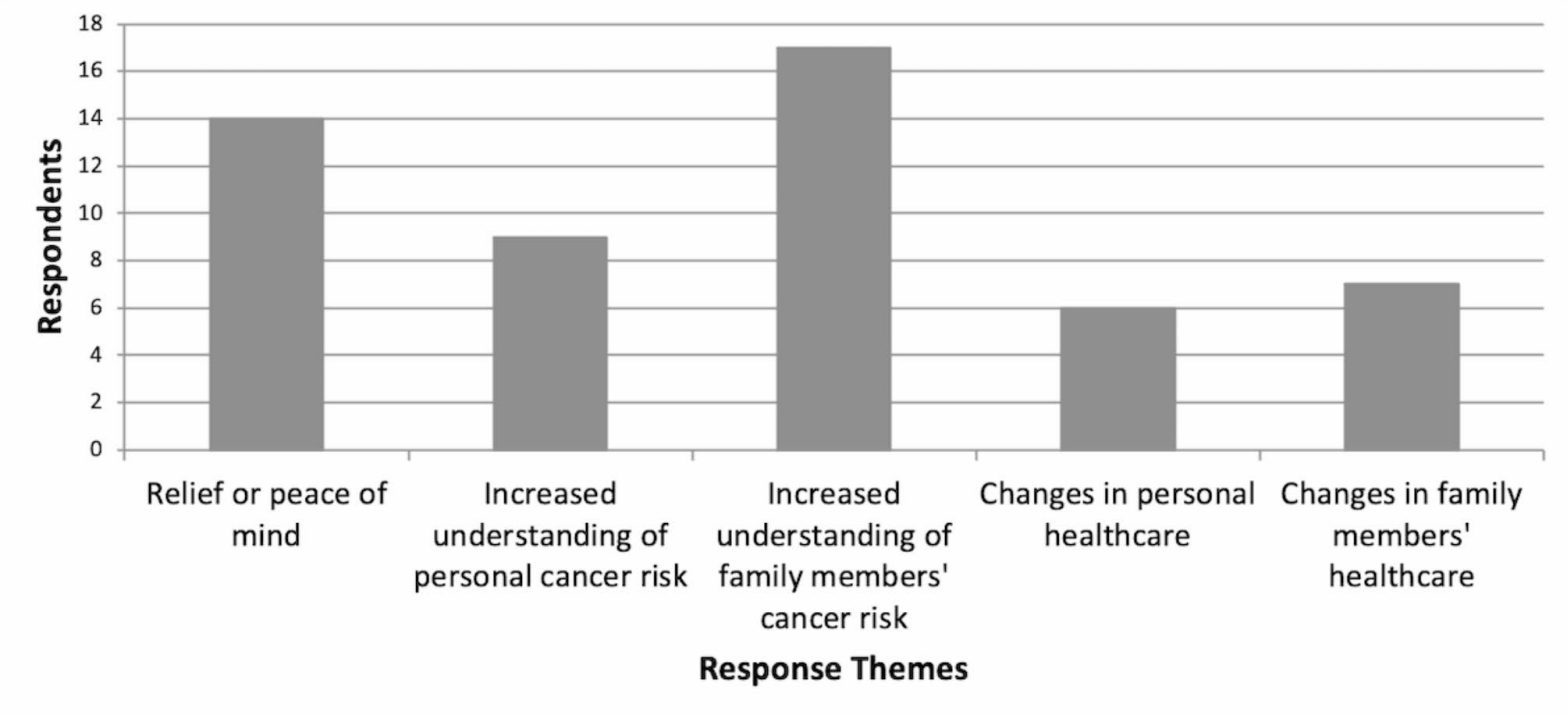
**Positive impacts of universal tumor screening.** Forty participants answered the question, “What were the positive effects, if any, of the universal screening policy on all colorectal cancer specimens for both you and your family?” Responses that did not fit a discernible theme or noted no benefit were omitted from this figure

Forty respondents described positive impacts of screening for themselves and their families. Their responses can be categorized into five themes: Relief or peace of mind, increased understanding of personal cancer risk, increased understanding of the family’s risk for cancer, changes in personal healthcare, and changes in family healthcare (Fig. 2). Many of the respondents whose screening results did not raise concern for Lynch syndrome based their responses on knowledge gained from the survey. In particular, feelings of relief were usually a reaction to understanding negative screening results. One woman’s daughter “thanked [her mother] for not ‘lynching’ her.” This response also demonstrated an understanding of familial risk for cancer. Some respondents interpreted the question more generally and responded with broad rather than personal positive impacts. One noted, “The positive would be that you are informed as a patient to any hereditary cancer concerns for yourself or your family.” Those who had genetic testing listed additional positive benefits. One individual with a Lynch-associated mutation mentioned their family had “increased awareness of health issues and proper testing/screening.” Another was more specific, noting that they and two relatives who tested positive received better screening while relatives who tested negative were “overjoyed to not have cancer hanging over their heads.” Another respondent noted as positive that the genetic counseling and testing process revealed a pathogenic variant in *BRCA2*. Those respondents who tested negative both noted feeling peace of mind.

Few participants listed any negative effects of screening on themselves or their families. One individual who had a negative screen result was concerned about the possibility of an out-of-pocket cost. The negative impacts were otherwise limited to those with a positive screen: one person was concerned about the wait for results and another noted that family members had suffered from depression after also being diagnosed with Lynch syndrome.

## Discussion

As expected, most respondents had not previously heard of Lynch syndrome or known about universal screening. The majority of respondents were in favor of universal screening without informed consent. Of those who were opposed, most were concerned about the lack of consent. A majority of respondents who screened positive and were contacted pursued genetic counseling. Those who declined genetic counseling did so due to being overwhelmed and having concerns about the costs involved. This is consistent with previous studies that have indicated colorectal cancer patients are interested in learning more about genetic risks [12, 22], and it supports the universal screening program as effective for the most part and acceptable to the patient population.

While the direct benefits of universal screening are associated with those who screen positive and their families, respondents who screened negative listed benefits of universal screening related to learning from this study that their results did not raise concern for Lynch syndrome. These included feelings of relief and increased understanding of familial cancer risks, which they would not have if not informed of their screening results. Respondents may have recognized this, as more than 90% of them preferred that everyone whose tumor is screened receive results. However, they may also have had some confusion regarding the difference between universal screening and genetic testing, or may not have understood that screening negative does not entirely rule out Lynch syndrome nor that close family members still have an increased risk for colorectal cancer. Indeed, in a care-related telephone call following the study, a participant incidentally demonstrated confusion about the difference between universal screening and genetic testing to one of the authors. Any information sheet given to patients about universal screening must be written at a basic reading level and clearly explain the difference between a screening and a diagnostic test.

Based on responses, an average patient’s ideal experience with universal tumor screening might include the following: they receive information about screening either before a sample is taken or at the confirmation of a diagnosis of colon cancer. Their gastroenterologist or colorectal surgeon informs them of their screening result and if positive, they are offered genetic counseling to further clarify risks. Implementation, as proposed above, could be time-consuming in practice and complicated by questions regarding the difference between a screening test and diagnostic germline genetic testing. Communication, therefore, is imperative, and preferably as early as possible in the universal screening process so as to be most successful. Patients who are educated about the potential benefits of universal screening will likely be more engaged should they receive a positive screening result. This active participation will hopefully translate into more patients following through with a genetic counseling appointment to enhance their understanding about the potential benefits of germline genetic testing to help clarify their screening results for both themselves and their family members, potentially leading to better uptake of genetic testing within the family.

Despite societal guidelines recommending widespread implementation of universal screening for Lynch syndrome, a number of studies have highlighted some of the real-world challenges encountered. One retrospective study looked at universal screening for Lynch syndrome compared with pedigree-based screening over 10 years in a tertiary hospital in Korea [23]. Their findings demonstrated that tumor testing was more effective than pedigree-based screening. However, use of either strategy alone led to some missed diagnoses, leading them to conclude that a combination of both methods would result in a more comprehensive ‘universal’ screening outcome. Another retrospective study analyzed the results of universal screening of patients with colorectal cancer at a referral hospital in Japan which led to the identification of only 2 probands with Lynch syndrome out of 463 (0.4%) patients screened, with only 8 out of 18 (44.4%) patients with positive screening results undergoing genetic testing [24]. Their study further questioned the implementation of routing screening in regions with low prevalence of Lynch syndrome, suggesting that the number of genetic testing candidates could be enriched by focusing on young-onset colorectal cancer cases. As a result of these challenges inherent with universal tumor screening for Lynch syndrome, not to mention the sequence of several screening tests which it entails (IHC followed in some cases by BRAF mutation analysis and/or MLH1 hypermethylation), in addition to the well-known challenges with family history collection, some have advocated for changing the paradigm to include upfront tumor sequencing [25] or universal germline genetic testing in colorectal cancer [26, 27].

This survey was framed around universal screening as a method of identifying Lynch syndrome in order to decrease the personal and societal impact of Lynch-associated cancers, as this had been the primary purpose of screening. However, physicians now also use knowledge of MSI and MMR deficiency to make treatment decisions, including eligibility for immune checkpoint inhibitor therapy. For example, a small study by Cercek et al. demonstrated that PD-1 blockade in mismatch repair-deficient, locally advanced rectal cancer resulted in an impressive clinical response rate such that patients were able to avoid chemoradiotherapy and surgery without disease progression or recurrence during a median follow-up of 12 months. The authors pointed out, however, that longer follow-up was needed to assess the duration of the response [28]. Given that some treatment decisions may now be informed by universal screening of the tumor, patients’ concerns about the screening process may be moderated somewhat. Tumor testing that can identify or raise concern for a hereditary cancer syndrome is now regularly performed for many different cancers in order to direct treatment. For example, identification of a somatic pathogenic variant in the *BRCA1* or *BRCA2* genes following tumor profiling may lead to personalized treatment with a PARP inhibitor [29], but it may also be indicative of Hereditary Breast and Ovarian Cancer syndrome if present in the germline [30, 31]. A single-arm prospective study using cisplatin monotherapy in the neoadjuvant setting reported a pathologic complete response rate of 61% among *BRCA* carriers, most of whom had *BRCA1* mutations and triple-negative breast cancer [32].

Studies have explored universal sequencing of tumors in colon cancer [33] but, most recently, evidence has begun to mount for universal germline testing in colorectal cancer. Universal germline testing identifies Lynch syndrome at a higher rate than universal screening protocols [34] and can identify other clinically actionable findings [35, 36]. Accordingly, NCCN Guidelines^®^ were updated in 2022 to suggest that germline testing may be considered for individuals with colon cancer regardless of age of diagnosis or family history [5].

If universal germline testing or even some types of tumor testing would be performed, one could argue that informed consent would be necessary [37], which would in turn alleviate the most significant concern among survey respondents who did not support the present process of implementing universal screening described herein. Universal germline testing, in particular, also removes the potential for a patient to be confused by the distinction between screening and diagnostic testing. It would, however, significantly increase the number of patients receiving genetic testing, even if some patients did decline. Given the small size of the genetics workforce and the growing number of indications for which genetic testing is now recommended, such as all breast cancers per the American Society of Breast Surgeons [38], alternatives to the traditional model of pre-test and post-test counseling are greatly needed. One option is for the treating provider to coordinate the genetic testing upfront, prior to referral to a genetics provider. This “mainstreaming” approach to testing, has been shown to increase uptake of genetic testing amongst patients with cancer diagnoses [39, 40]. Genetic counselors can partner with these ordering providers to choose a standard panel of appropriate genes, create patient-friendly consent forms and information sheets like those endorsed by our survey respondents, and provide post-test counseling either for all or a subset of patients. In an alternative model pioneered in patients with ovarian or pancreatic cancer, a genetic counseling assistant embedded in the cancer clinic provides video-based education and obtains consent for genetic testing, personal and family history details, and a sample for testing, with the genetic counselor receiving results and providing post-test counseling [41]. In either approach, including genetic testing as an integrated part of their care can help reduce patients’ feelings of being overwhelmed by or concerned about the costs of an extra appointment, which were reasons survey respondents gave for not pursuing genetic counseling and testing under the current universal screening model.

As the field moves toward universal genetic testing for individuals with colorectal cancer, or possibly patients with any solid tumor diagnosis [42, 43, 44], studies regarding patient understanding, satisfaction, and personal experiences, such as the one described herein, will help inform implementation of these new processes. While informed consent will be necessary with germline testing, the feedback of the individuals surveyed in our study can help direct that implementation and help anticipate the concerns of future participants.

### Study limitations

The survey is limited in its scope by its respondents, and especially by the 22% response rate. This may be a result of several factors, such as the 17-page length of the survey or that any individuals whose tumors screened negative had no prior relationship with the cancer genetics program. 95% of those who did not respond were individuals who screened negative, while individuals who chose to pursue genetic counseling were significantly overrepresented, with 11 of 19 individuals who went through that process during the study period responding to the survey (*p* = .0051). In addition, a significantly disproportionate number of respondents were female, with 42 women and 23 men responding compared with 156 men and 141 women to whom the survey was sent (*p* = .0020). However, support for universal screening did not vary significantly either by sex (*p* = 1) or by whether a person had genetic counseling (*p* = .27).

Several participants answered questions on the paper copy of the survey that they should not have if they had read and understood the instructions for the survey. These answers were disregarded because if those respondents had completed the survey online, they would not have viewed those questions due to the branching logic used in REDCap. Others checked multiple answers when instructed to answer only one; these responses were consolidated as “Other.” These occurrences, however, suggest that some participants were not fully engaged with or may have misunderstood the survey. It is possible that the $10 gift card offered as compensation for time spent taking the survey influenced some participants who might not otherwise have participated. These individuals may have sped through the survey for the gift card and not carefully considered their responses. On the other hand, however, the $10 gift card may not have been sufficient compensation for the time required to adequately answer the 17-page survey, providing another possible explanation for the 22% survey response.

## Conclusions

Past studies have shown that universal screening of colorectal tumors can identify individuals and families with Lynch syndrome and promote care that decreases the overall burden of Lynch-associated cancers. Effective implementation with good communication regarding the process is essential to the success of universal screening. Patient support and satisfaction and the response of patients whose screening results raised concern for Lynch syndrome are important facets of that success. Respondents to this survey, representing 22% of total colorectal cancer patients within the first 30 months under the universal screening policy at Penn State Hershey Medical Center, were mostly in favor of universal screening. However, many would prefer to be better informed about the policy and their own screening results. Based on these findings, the implementation of a universal screening policy must strike a balance between patient preferences, the person-hours required to accommodate those preferences, and the ability to provide the best medical care. Further research into effective implementation of universal screening at other health care facilities, and of other tumor types, can help elucidate that balance.

Since this study was conducted, the focus of universal screening has shifted to informing treatment beyond surgical decisions, such as the use of immunotherapy with pembrolizumab. Genetic testing of other tumor types to personalize treatment has become more commonplace and universal germline testing is on the horizon. As the landscape of universal screening and testing continues to evolve, so will patient preferences and acceptance. It is therefore essential that patient understanding, preferences, and satisfaction continue to be assessed as the types of screening and testing performed change and criteria for inclusion expand.

## Data Availability

The datasets used and/or analyzed during the current study are available from the corresponding author on reasonable request.

## Abbreviations

MSI: Microsatellite Instability
IHC: Immunohistochemistry
MMR: Mismatch Repair
NCCN: National Comprehensive Cancer Network
VUS: Variant of Uncertain Significance
